# Impact of ε and θ subunits on pharmacological properties of α3β1 GABA_A _receptors expressed in *Xenopus *oocytes

**DOI:** 10.1186/1471-2210-6-1

**Published:** 2006-01-13

**Authors:** Martin Ranna, Saku T Sinkkonen, Tommi Möykkynen, Mikko Uusi-Oukari, Esa R Korpi

**Affiliations:** 1 Institute of Biomedicine, Pharmacology, Biomedicum Helsinki, POB 63 (Haartmaninkatu 8), FI-00014 University of Helsinki, Finland; 2 Department of Pharmacology and Clinical Pharmacology, University of Turku, Itäinen Pitkäkatu 4, FI-20520 Turku, Finland

## Abstract

**Background:**

γ-Aminobutyric acid type A (GABA_A_) receptors provide the main inhibitory control in the brain. Their heterogeneity may make it possible to precisely target drug effects to selected neuronal populations. *In situ *hybridization using rat brain sections has revealed a unique expression of GABA_A _receptor ε and θ subunit transcripts in the locus coeruleus, where they are accompanied at least by α3, α2, β1 and β3 subunits. Here, we studied the pharmacology of the human α3β1, α3β1ε, α3β1θ and α3β1εθ receptor subtypes expressed in *Xenopus *oocytes and compared them with the γ2 subunit-containing receptors.

**Results:**

The GABA sensitivites and effects of several positive modulators of GABA_A _receptors were studied in the absence and the presence of EC_25 _GABA using the two-electrode voltage-clamp method. We found 100-fold differences in GABA sensitivity between the receptors, α3β1ε subtype being the most sensitive and α3β1γ2 the least sensitive. Also gaboxadol dose-response curves followed the same sensitivity rank order, with EC_50 _values being 72 and 411 μM for α3β1ε and α3β1γ2 subtypes, respectively. In the presence of EC_25 _GABA, introduction of the ε subunit to the receptor complex resulted in diminished modulatory effects by etomidate, propofol, pregnanolone and flurazepam, but not by pentobarbital. Furthermore, the α3β1ε subtype displayed picrotoxin-sensitive spontaneous activity. The θ subunit-containing receptors were efficiently potentiated by the anesthetic etomidate, suggesting that θ subunit could bring the properties of β2 or β3 subunits to the receptor complex.

**Conclusion:**

The ε and θ subunits bring additional features to α3β1 GABA_A _receptors. These receptor subtypes may constitute as novel drug targets in selected brain regions, e.g., in the brainstem locus coeruleus nuclei.

## Background

The A-type receptors for γ-aminobutyric acid (GABA_A_) mediate the majority of fast inhibitory transmission in the mammalian central nervous system. Many clinically important drugs including benzodiazepines, barbiturates and general anesthetics act as positive allosteric modulators of this receptor [1-3]. GABA_A _receptors are pentameric ligand-gated ion-channels, composed of subunits from many subunit classes: α1–6, β1–3, γ1–3, δ, ε, θ, π, and ρ1–3. This produces a great heterogeneity in the receptor structures and receptor properties. The main receptor population containing the α1 subunit is responsible for only some, but not all, behavioral and physiological effects of GABA_A _receptors and, most importantly, the animals devoid of the α1 subunits survive without dramatic abnormalities [4-6]. Therefore, it is possible that minor subtypes are also relevant in physiology and as pharmacological targets.

Here, we focused our study on minor populations of novel ε and θ subunit-containing GABA_A _receptors. These subunits are enriched in several discrete nuclei, most remarkably in the bilateral brainstem nucleus locus coeruleus (LC) [7]. The ε and θ subunit genes are clustered with the α3 subunit gene in the chromosome Xq28 [1], suggesting a common ancestry with the three other β-α-γ GABA_A _receptor subunit gene clusters in other chromosomes, although the θ and ε subunits have diverged remarkably from β- and γ-like sequences [8,9]. The *in situ *hybridization signals of rat brain sections have shown that the LC neurons express among the α subunits, the α3 and α2 subunit mRNAs, but not those of α1, α4, α5 or α6 [7,10-12], among the β subunits, the β1 and β3 subunit mRNAs [13], but none of the γ1–3 mRNAs are clearly enriched in the LC neurons. In contrast to the expression profiles of the most GABA_A _receptor subunits, the ε and θ subunit mRNAs are strongly enriched in the LC neurons [12]. These expression profiles suggest a likely existence at least of α3β1θ, α3β1ε and α3β1 receptor populations among the LC GABA_A _receptors, with additional roles of α2 and β3 subunits in either replacing the α3 and β1 and/or perhaps co-assembling with them (see [14]).

The θ subunit has the highest sequence identity (about 50%) with the β1 subunit [15]. It should hypothetically form functional receptors with α and β or α and γ subunits, but in practice it may not form functional receptors when coexpressed with an α subunit or an α and β subunit as the αβθ combination failed to respond to GABA [15]. Thus, only little is known about the pharmacological properties of the θ subunit, and its role has not been satisfactorily studied in different receptor subunit combinations.

The ε subunit is more related to the γ subunits [16,17]. The effect of the ε subunits on the properties of GABA_A _receptor subtypes has been studied only in a few receptor subtypes, mostly in α1 subunit-containing receptors. The ε subunit-containing receptors display insensitivity to benzodiazepines and reduced efficacy for anesthetics, spontaneous channel openings, sensitivity to picrotoxin, rapid or more extensive desensitization, and this subunit has abolished the outward rectification in recombinant receptors [17-20].

The LC is the largest noradrenergic nucleus with many projections innervating major levels of neuraxis. It plays an important role in the regulation of anxiety states, vigilance, attention and memory functions [21]. It has been shown that 50 % of the nerve terminals in the LC can accumulate [^3^H]GABA and that the LC neurons contain glutamic acid decarboxylase, the GABA biosynthetic enzyme [11,22]. The LC harbors a unique repertoire of GABA_A _receptors due to the expression of ε and θ subunits. This report describes a characterization of recombinant α3β1θ/ε receptors that most likely form major GABA_A _receptor populations in the LC neurons.

## Results

### GABA-site sensitivity of recombinant human GABA_A _receptors expressed in *Xenopus *oocytes

**Figure 1:**
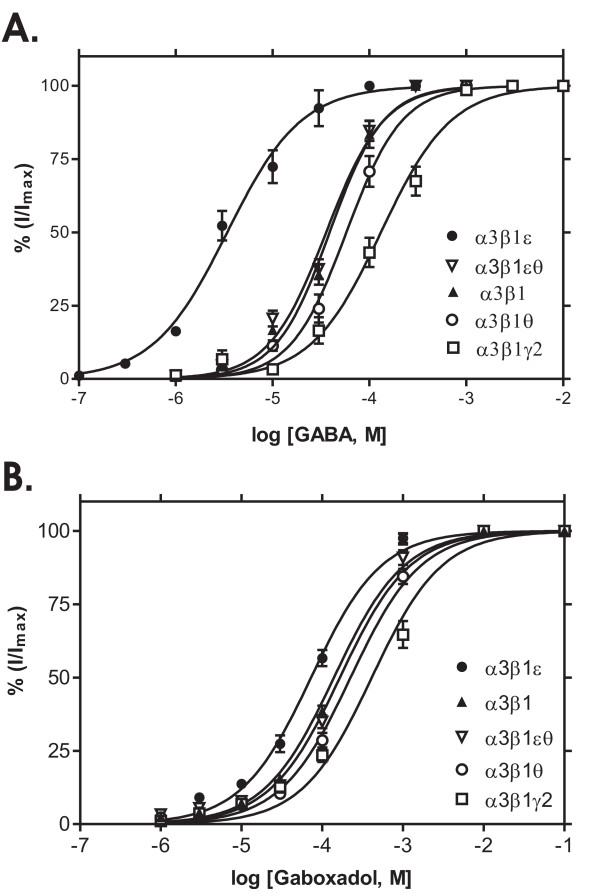
**Responses to GABA site ligands**. GABA and gaboxadol sensitivities of recombinant α3β1, α3β1γ2, α3β1θ, α3β1ε and α3β1θε GABA_A _receptors expressed in *Xenopus *oocytes. **A**. The values for currents induced by various GABA concentrations are given as means ± standard errors (*n *= 8 for α3β1, n = 6 for α3β1γ2, n = 7 for α3β1θ; n = 6 for α3β1ε and n = 7 for α3β1θε receptors). GABA dose-response curves were obtained by non-linear regression fits and GABA EC_50 _values were calculated for individual cells, being 55 ± 6 μM for α3β1 receptors, 200 ± 38 μM for α3β1γ2 (*p *< 0.01, unpaired *t*-test from α3β1), 2.3 ± 0.5 μM for α3β1ε (*p *< 0.001 from α3β1) and 81 ± 18 μM for α3β1θ (*p *> 0.05) and 35 ± 9 μM for α3β1θε receptors. Maximal currents elicited by GABA were 1.5 ± 0.9 μA for α3β1, 2.2 ± 0.5 μA for α3β1γ2, 1.2 ± 0.3 μA for α3β1θ; 1.2 ± 1.0 μA for α3β1ε and 0.9 ± 0.4 μA for α3β1θε combinations. **B**. Gaboxadol-induced currents are given as means ± standard errors (*n *= 7 for α3β1, n = 5 for α3β1γ2, n = 4 for α3β1θ; n = 5 for α3β1ε and n = 4 for α3β1θε receptors). Gaboxadol dose-response curves were obtained by non-linear regression fits and the EC_50 _values were calculated for individual cells, being 139 ± 19 μM for α3β1 receptors, 411 ± 13 μM for α3β1γ2 (*p *< 0.05, unpaired *t*-test from α3β1),72 ± 5 μM for α3β1ε (*p *> 0.05 from α3β1), 224 ± 20 μM for α3β1θ (*p *> 0.05) and 165 ± 19 μM for α3β1θε receptors. Maximal currents elicited by gaboxadol were 2.5 ± 0.9 μA for α3β1, 1.2 ± 0.5 μA for α3β1γ2, 0.55 ± 0.2 μA for α3β1θ, 1.0 ± 0.4 μA for α3β1ε and 0.8 ± 0.3 μA for α3β1θε combinations.

GABA elicited inward currents in α3β1, α3β1γ2s, α3β1θ, α3β1ε and α3β1εθ GABA_A _receptors that were well distinguished from the baseline, whereas no currents were detected in the α3θε combination or the dimer α3θ combination (data not shown). The GABA concentration-response curves are depicted in Fig. 1A. Currents were normalized to the maximal peak currents induced by GABA (I_max_). GABA concentrations producing 50 % of the maximum efficacy (EC_50_) varied significantly between the receptor subtypes. The α3β1ε receptors had the highest GABA sensitivity (EC_50 _2.3 μM), being 25-fold higher than in α3β1 and α3β1θ receptors and almost 100-fold higher than in α3β1γ2 receptors (200 μM). The GABA sensitivity of α3β1θ receptors was not different from that of α3β1, while the addition of ε subunit significantly increased GABA sensitivity and the addition of γ2 reduced it. When both ε and θ subunits were co-injected, the oocytes had similar EC_50 _values for GABA as the α3β1 receptors.

The potency of gaboxadol, a partial agonist of the GABA site [23], at all tested receptor subtypes was lower than that of GABA (Fig. 1B). Gaboxadol had the highest potency at the α3β1ε receptor (EC_50 _= 72 μM), followed by α3β1 (139 μM), α3β1εθ (165 μM), α3β1θ (224 μM) and α3β1γ2 receptors (411 μM). Since different batches of oocytes were used in Fig. 1, maximal currents induced by GABA and gaboxadol could not be compared. When 1 mM concentrations of GABA and gaboxadol were individually applied to the same oocytes, the gaboxadol-evoked currents were only about 50% of the corresponding GABA currents (data not shown), suggesting that gaboxadol is a partial agonist at these receptor subtypes. More specifically in α3β1ε receptors, in which 1 mM concentrations of both agonists saturate the receptor (Fig. 1), gaboxadol-induced currents were 34 ± 2 % of the GABA currents (n = 3) and both agonists applied simultaneously evoked a current that was 90 ± 1 % of the GABA current alone.

### ε subunit-containing receptors display leakage currents that can be blocked with picrotoxin

**Figure 2:**
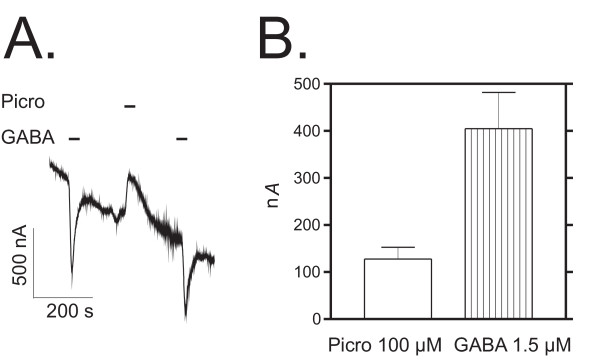
**Constitutive activity of ε subunit-containing receptors**. The ε subunit-containing receptors display spontaneous currents that can be blocked with picrotoxin. **A**. A representative current trace recorded from an oocyte expressing spontaneously open α3β1ε receptors is shown. GABA (30 μM) application induced typical inward currents. Picrotoxin (Picro, 100 μM) reversibly blocked spontaneously active receptors as shown by blockade (outward current) of apparent leakage current. **B**. Peak amplitudes (in nA given as means ± standard errors) of GABA- and picrotoxin-induced currents in six oocytes expressing α3β1ε receptors.

Recombinant α3β1ε receptors exhibited spontaneous activity, which could be seen by outward currents during application of 100 μM picrotoxin, the GABA_A _receptor channel blocker, and often by the continuous drift of the baseline. Figure 2 illustrates the effect of picrotoxin (panel A) and compares its amplitude to the inward currents induced by 1.5 μM GABA (panel B).

### θ subunit confers high efficacy for etomidate

**Figure 3:**
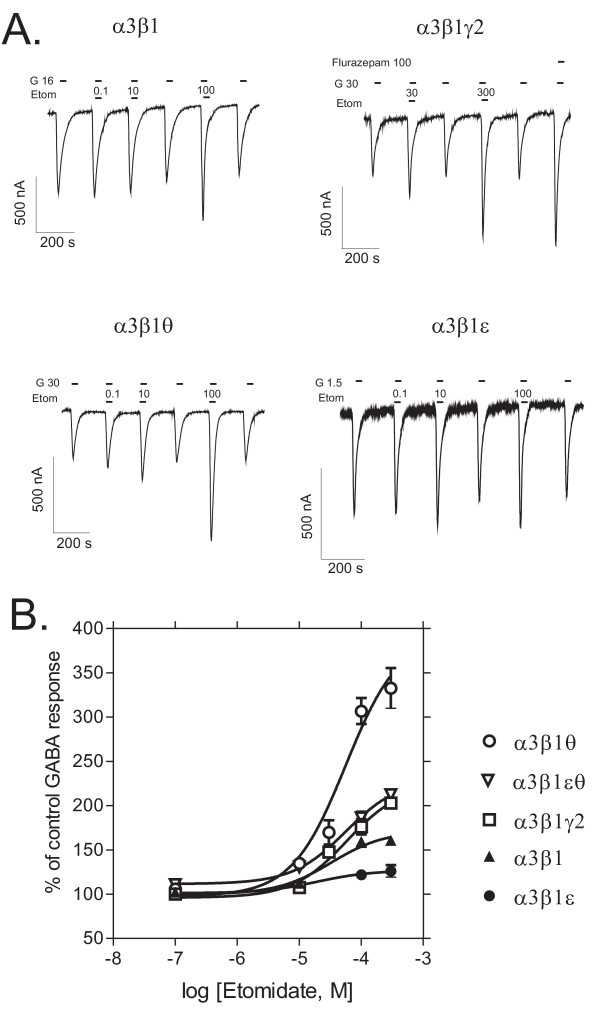
**Sensitivities to the anesthetic etomidate**. Modulation of GABA-induced currents by etomidate in recombinant GABA_A _receptors expressed in *Xenopus *oocytes. **A**. Representative traces of etomidate (*Etom*, μM) modulation of GABA-induced currents (*G*, EC_25 _in μM) in different receptor subtypes. Also the effect of flurazepam at 100 μM is shown for the α3β1γ2 receptors. **B**. Etomidate dose-response curves in the presence of EC_25 _GABA (set to 100 %). The values are means ± standard errors (n = 6 for α3β1; n = 7 for α3β1γ2; n = 9 for α3β1θ; n = 11 for α3β1ε and n = 4 for α3β1θε receptors).

In the presence of 100 μM etomidate, GABA-induced currents were 159 ± 6 % of the control GABA response for α3β1 (mean ± standard error, n = 5), 176 ± 9 % for α3β1γ2 (n = 5), 298 ± 18 % for α3β1θ (n = 6), 122 ± 5 % for α3β1ε (n = 10) and 186 ± 7 % for α3β1εθ (n = 4) receptors (Fig. 3), indicating that the α3β1θ receptors were potentiated by etomidate the most (*p *< 0.001, Dunnett's test) and the α3β1ε receptors the least (*p *< 0.05). Nonlinear regression fit of etomidate potentiation of GABA-induced currents revealed that etomidate had higher efficacy in α3β1θ than α3β1γ2 receptors (maximal potentiation 364 ± 43 vs. 222 ± 15 % of the control GABA response, respectively, *p *< 0.01, unpaired *t*-test), but there was no drastic difference in etomidate potency (EC_50 _~ 50 μM in both combinations). In α3β1εθ receptors, the efficacy of etomidate was clearly reduced from that for α3β1θ receptors.

### θ and γ2 subunits enhance propofol efficacy

**Figure 4:**
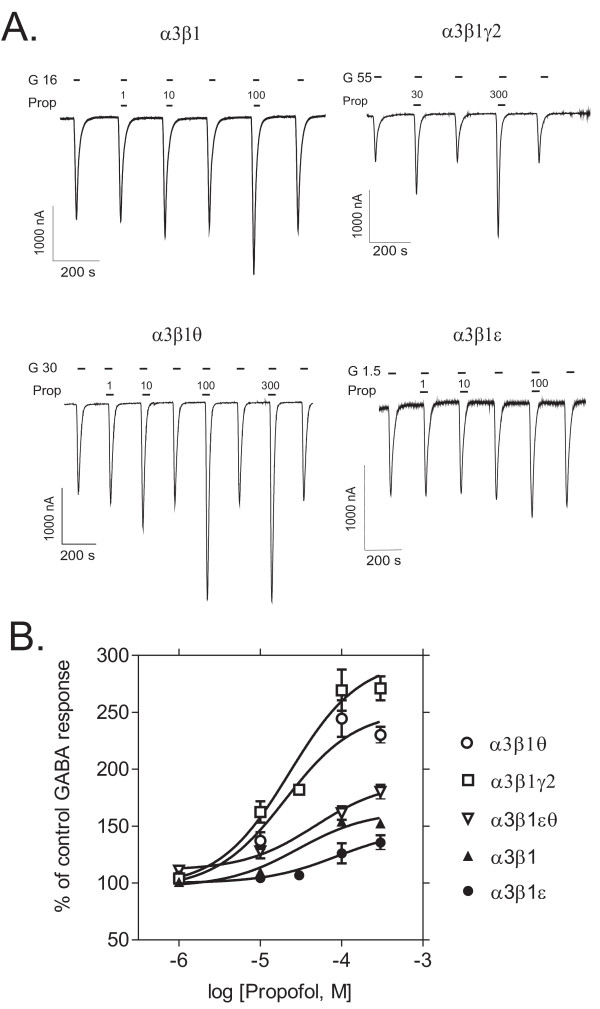
**Sensitivities to the anesthetic propofol**. Modulation of GABA-induced currents by propofol in recombinant GABA_A _receptors expressed in *Xenopus *oocytes. **A**. Representative traces of propofol (*Prop*, μM) modulation of GABA-induced currents (*G*, EC_25 _in μM) in different receptor subtypes. **B**. Propofol dose-response curves in the presence of EC_25 _GABA (set to 100 %). The values are means ± standard errors (n = 6 for α3β1; n = 9 for α3β1γ2; n = 6 for α3β1θ; n = 7 for α3β1ε and n = 3 for α3β1θε receptors).

In the presence of 100 μM propofol, GABA-induced currents were 153 ± 4 % of the control GABA response for α3β1 (mean ± standard error, n = 5), 269 ± 18 % for α3β1γ2 (n = 7), 244 ± 16 % for α3β1θ (n = 5), 126 ± 9 % for α3β1ε (n = 6) and 161 ± 6 % for α3β1εθ (n = 3) receptors (Fig. 4), indicating that both γ2 and θ subunit-containing receptors were more potentiated by propofol than α3β1 combination (*p *< 0.001 for both combinations vs. α3β1, Dunnett's test). The α3β1ε receptors were hardly potentiated at all, but addition of the θ subunit to this combination increased the efficacy. Nonlinear regression fit of propofol potentiation of GABA-induced currents yielded a similar potency (EC_50 _= 20 μM) and maximal potentiation for α3β1γ2 and α3β1θ receptors (297 ± 15 and 257 ± 12 %, respectively).

### ε and θ subunits reduce neurosteroid potency

**Figure 5:**
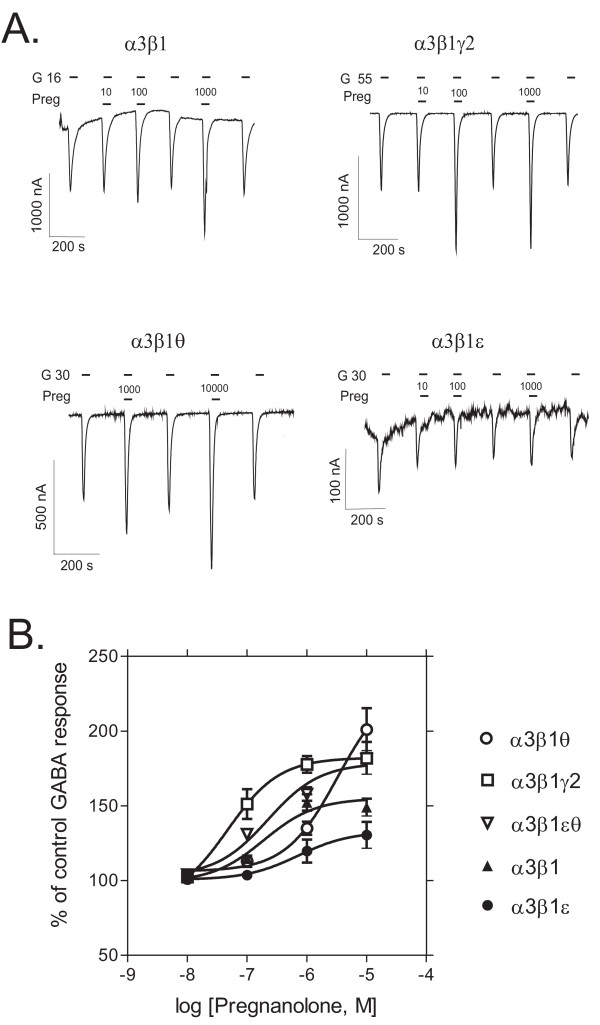
**Sensitivities to the neurosteroid pregnanolone**. Modulation of GABA-induced currents by the neurosteroid pregnanolone in recombinant GABA_A _receptors expressed in *Xenopus *oocytes. **A**. Representative traces of pregnanolone (*Preg*, nM) modulation of GABA-induced currents (*G*, EC_25 _in μM) in different receptor subtypes. **B**. Pregnanolone dose-response curves in the presence of EC_25 _GABA (set to 100 %). The values are means ± standard errors (n = 6 for α3β1; n = 5 for α3β1γ2; n = 5 for α3β1θ; n = 6 for α3β1ε and n = 4 for α3β1θε receptors).

In the presence of 1 μM pregnanolone, GABA-induced currents were 152 ± 5 % of the control GABA response for α3β1 (mean ± standard error, n = 6), 177 ± 6 % for α3β1γ2 (n = 5), 135 ± 4 % for α3β1θ (n = 5), 119 ± 8 % for α3β1ε (n = 6) and 158 ± 4 % for α3β1εθ (n = 4) receptors, indicating that γ2 subunit-containing receptors were slightly more potentiated by pregnanolone than α3β1 receptors (*p *< 0.05, Dunnett's test). Nonlinear regression fit of pregnanolone potentiation of GABA-induced currents yielded remarkable differences in pregnanolone potency as α3β1γ2 receptors were highly sensitive and robustly potentiated by pregnanolone [EC_50 _47 nM and maximal potentiation 182 ± 10 % (Fig. 5)]. The α3β1ε, α3β1 and α3β1θ receptors were apparently much less sensitive (α3β1 receptors had EC_50 _of 190 nM and in θ and ε subunit-containing receptors it could not be accurately estimated due to lack of saturation). The α3β1 and α3β1θ receptors were clearly potentiated, but there was only little neurosteroid potentiation in α3β1ε receptors. Interestingly, the addition of both ε and θ subunits appears to increase the potency and reduce the efficacy as compared to α3β1θ receptors.

### ε and θ subunits do not confer benzodiazepine sensitivity nor affect barbiturate modulation

**Figure 6:**
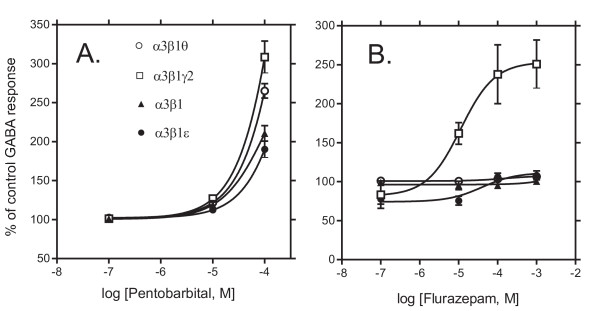
**Sensitivities to the barbiturate pentobarbital and the benzodizepine flurazepam**. Modulation of GABA-induced currents by pentobarbital and flurazepam in recombinant GABA_A _receptors expressed in *Xenopus *oocytes. **A**. Pentobarbital dose-response curves in the presence of EC_25 _GABA (set to 100 %). The values are means ± standard errors (n = 6 for α3β1; n = 9 for α3β1γ2; n = 4 for α3β1θ and n = 8 for α3β1ε receptors). **B**. Flurazepam dose-response curves in the presence of EC_25 _GABA (set to 100 %). The values are means ± standard errors (n = 6 for α3β1; n = 9 for α3β1γ2; n = 4 for α3β1θ and n = 8 for α3β1ε receptors).

Benzodiazepines presumably bind to the interface between α and γ2 subunits [24]. In the presence of 1 mM flurazepam, GABA-induced currents were affected as follows: 100 ± 3% of the GABA control response (mean ± standard error, *n *= 4) in α3β1, 251 ± 31% in α3β1γ2 (*n *= 4), 107 ± 3% in α3β1θ (*n *= 3) and 108 ± 6% in α3β1ε (*n *= 5) receptors. As expected [25], benzodiazepine sensitivity was achieved only among the receptor subtype containing the crucial γ2 subunit. Nonlinear regression fit of flurazepam effects on GABA-induced currents revealed EC_50 _= 11.4 μM and maximal potentiation of 254 ± 21% of the GABA control response in α3β1γ2 receptors (Fig. 6). Possible negative modulation of ε subunit-containing receptors at low flurazepam concentrations was not statistically significant (*p *> 0.05, n = 6).

In the presense of 100 μM pentobarbital, GABA-induced currents were 210 ± 10 % of the control GABA response in α3β1 (mean ± standard error, n = 5), 308 ± 20 % in α3β1γ2 (n = 9), 265 ± 9 % in α3β1θ (n = 4) and 190 ± 10 % in α3β1ε (n = 8) receptors, showing that γ2 subunit-containing receptors were more efficaciously potentiated by pentobarbital than other combinations (*p *< 0.05, ANOVA), which did not differ from each other.

### Direct activation by etomidate and propofol

**Figure 7:**
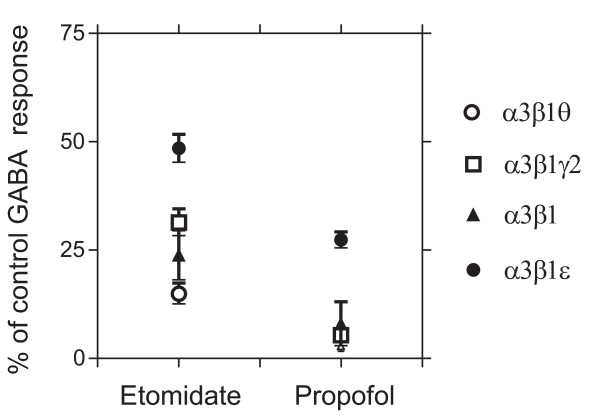
**Direct receptor activation by etomidate and propofol**. Efficacy of a high concentration (300 μM) of etomidate and propofol in provoking receptor currents as compared to control EC_25 _GABA responses (set to 100 %). The values are means ± standard errors (n = 5 for α3β1; n = 6 for α3β1γ2; n = 5 for α3β1θ and n = 7 for α3β1ε receptors).

Anesthetics at high concentrations are known to open the GABA_A _receptor ionophores also directly in the absence of GABA, i.e., without allosteric interaction with the GABA binding sites [1]. We used etomidate and propofol at high 300 μM non-saturating concentrations, and found that the α3β1ε, α3β1γ2, α3β1 and α3β1θ receptors were all slightly activated in the absence of GABA (Fig. 7). Etomidate at 300 μM provoked currents from 14.5 % (α3β1θ, n = 4) up to 46.8% (α3β1ε, n = 4) of EC_25 _GABA control responses. Propofol at 300 μM provoked even smaller currents ranging between 3 % (α3β1θ, n = 5) to 25 % (α3β1ε) of the EC_25 _GABA responses. All drugs and GABA controls were applied to each oocyte. These results indicate that the effects of etomidate and propofol in the presence of EC_25 _GABA are not due to direct effects on the receptor channel, but rather due to allosteric potentiation of the GABA action.

## Discussion

The present data indicate that the locus coeruleus -enriched ε and θ subunits of GABA_A _receptors produce significant functional and pharmacological changes on the α3β1 receptors. These features also differentiate them from γ2 subunit-containing receptors. It is thus possible that the novel subunits may form receptor populations that could be selectively targeted by future drugs.

As compared to the α1 subunit-containing receptors, the α3 subunit receptors display a reduced sensitivity to GABA, which has been traced to an extracellular N-terminal domain of four amino acids within the α subunits that mediates the distinct sensitivities to GABA [26]. We confirmed here the very low GABA sensitivity in most of the α3 subunit combinations tested (Fig. 1A), the EC_50 _values for GABA being 10–100 times higher than those found in previous studies in recombinant α1 and especially α6 subunit-containing receptors expressed in oocytes [27]. However, the ε subunit confers high GABA sensitivity to the receptors, the EC_50 _for GABA being almost 100-fold lower than that for α3β1γ2 receptors. Thus, the ε subunit makes the α3-receptors as sensitive to the natural transmitter as most other receptor subtypes are. This effect of ε subunit may be specifically dependent on the α subunit, since in α1 subunit-containing receptors the addition of ε does not alter GABA sensitivity [28]. The high GABA potency of the α3β1ε receptors was apparently reduced when θ subunit was added. However, whether all subunits were present in the same pentameric receptors in our conditions remains uncertain and awaits further experiments with linked subunits.

Previously it has been shown that gaboxadol binds to and acts on all GABA_A _receptors with a relatively low selectivity [29,30]. More recently, gaboxadol has been suggested to affect more efficiently or potently extrasynaptically located GABA_A _receptors (perhaps α4/5/6βδ subtypes) [31-34], which are found at high concentrations in the hippocampus, thalamus and cerebellum, but are also present widely in the neocortex [35]. Gaboxadol potency and efficacy are dependent on the α subunit [36,37]. In the present study, gaboxadol had a low potency at all α3 subunit-containing receptors, but its potency was higher in the presence of ε subunits than with γ2 subunits (Fig. 1B). However, the potency variation was smaller between the subtypes with gaboxadol than with GABA, which might have something to do with the differences in intrinsic efficacies. We found high apparent efficacies for gaboxadol in most receptor subtypes (Fig. 1), but in comparison to GABA in the same oocytes, its efficacy was lower than that of GABA at 1 mM concentrations. This is consistent with partial agonism of gaboxadol that has previously been shown for α3β1/2/3γ2 receptors [36].

A feature of the ε subunit-containing receptors was the spontaneous activity of the chloride channel, which has been observed in other recombinant receptor experiments when ε is expressed with α1 and β subunits [28,38,39]. The ε subunit-containing receptors have been suggested to have higher stability of the energetically more favorable ionophore open-state position than closed-state position, functionally causing "leakage" currents [38]. The spontaneously active currents can be blocked by GABA_A _antagonists such as picrotoxin (Fig. 2) [39,40]. In line with our results with α3β1 receptors, agonist sensitivity has been shown to be increased with the degree of spontaneous receptor current in α1β2γ2/ε receptors ([40], but see [28]). The role of constitutive receptor channel activity is still uncertain, since e.g. Sergeeva and Haas [41] found that constitutive activity did not correlate with the expression of ε subunit mRNA in cultured hypothalamic neurons. Thompson et al. [19] have suggested that the relative level of ε subunit expression may influence the emergence of constitutive channel activity. At least in recombinant receptors *in vitro*, the constitutive activity is the hallmark of ε subunit-containing receptors.

In our experiments, the ε-containing receptors were mildly, if at all, modulated by different allosteric agonists (flurazepam, pregnanolone, etomidate and propofol) in the presence of GABA, supporting the previous findings about benzodiazepine-insensitivity and reduced efficacy of general anesthetics at ε subunit-containing receptors [17]. Since we applied GABA and the modulators simultaneously, part of the response at high modulator concentrations could be due to their direct effects. However, etomidate and especially propofol alone at 300 μM concentration caused only small responses in the absence of GABA, indicating that the main effect of these modulators was the potentiation of GABA action. Spontaneous openings induced by the ε subunit may decrease the anesthetic efficacy compared to that in α3β1 dimer subtype, although the baseline GABA sensitivity differences were taken into account by adjusting the GABA concentrations to EC_25 _of each receptor subtype. Furthermore, the addition of ε subunit even to the α1β3θ receptors that show high etomidate and propofol efficacies, reduced the efficacies of these drugs. Only the barbiturate pentobarbital retained its activity in the ε subunit-containing receptors in our study. Our results are in concert with those of Whiting et al. [42] and Neelands et al. [38], who found barbiturate potentiation in α1β1ε and α1β3ε combinations expressed in oocytes. However, Irnaten et al. [43] found the cardiac parasympathetic neurons transfected with the ε subunit to lose their pentobarbital modulation of spontaneous IPSCs and pentobarbital-evoked facilitation of GABAergic currents. It should be noted that the LC neurons from the rat are sensitive to barbiturates using intracellular recording from *in vitro *brain slices [44], indicating that the expression of ε subunits in these neurons does not make the neurons insensitive to barbiturates.

The θ subunit was clearly more silent in various receptor subunit combinations than the ε subunit. GABA sensitivity was not altered by inclusion of the θ subunit in α3β1 receptors. Injections of the oocytes with α3 and θ or α3, θ and ε subunit cRNA cocktails did not produce functional responses to GABA, although the amino acid sequence identity of the θ subunit resembles that of the β subunits [15]. This indicates that the θ subunit fails to fully replace the β subunits. Pharmacologically the θ subunit brought about clear properties to α3β1θ receptors, the most striking being the increased efficacy of the anesthetic etomidate (Fig. 3). This is important, since etomidate belongs to the growing number of GABA_A _receptor ligands the efficacy of which is largely determined by residues in the second transmembrane region of the β subunits [1]. Etomidate is more potent and efficacious on β2 and β3 receptors than on β1 receptors [45,46], which apparently explains its low efficacy in our α3β1 and α3β1γ2 receptors. Our experiments should be extended to the amino acid residue level by testing whether the residue in the θ subunit (Gln), homologous TM II region residue Ser265 in the β1 subunit vs. Asn265 in the β2 and 3 subunits [46], is responsible for the altered pharmacology. This might reveal the molecular mechanisms of how θ subunits promote the actions of etomidate in β1 subunit-containing receptors. Our finding might suggest that even β1 subunit-containing receptors may be partially responsible for the actions of etomidate, when the receptor also harbors the θ subunit. This could complicate the conclusions recently drawn from the studies with mouse lines having etomidate-insensitive Asn-Ser point-mutated β2 or β3 subunits, with the β2 subunit-containing receptors selectively mediating etomidate sedation and hypothermia and β3 subunit-containing receptors etomidate hypnosis and anesthetic immobility [47-50]. However, since the θ subunit increased only the efficacy of etomidate and failed to provide the higher potency that β2 and β3 do, the β1θ subunit-containing receptors might not have a great influence on the *in vivo *sensitivity to etomidate.

Propofol efficacy was also high in the presence of the θ subunit, but now identical to that of the γ2 subunit. Similar efficacy profile was found for the neurosteroid pregnanolone, but not so clearly for pentobarbital. The θ subunits are not critical for the actions of benzodiazepines, as the GABA potentiation by flurazepam did not take place in α3β1θ receptors.

## Conclusion

Locus coeruleus neurons express a rare repertoire of GABA_A _receptor subunits to form GABA_A _receptor subtypes with specific characteristics. Our present data suggest that α3β1θ and α3β1ε receptor subtypes, expressed uniquely in the locus coeruleus, are differentially affected by benzodiazepines and anesthetics in comparison to α3β1 and α3β1γ2 receptors. This pharmacological diversity might be exploited to find subtype-selective compounds for the treatment of stress and anxiety, and for the induction of analgesia and sedation. Furthermore, the ε subunit confers high transmitter sensitivity, which may make it a dominant site for physiological modulation, especially if located in receptors outside synapses.

## Methods

All experimental procedures were approved by the Institutional animal use and care committee of the University of Helsinki.

### Recombinant receptor expression in *Xenopus *oocytes

Capped cRNAs encoding for human GABA_A _receptor subunits α3, β1, γ2S, ε and θ were transcribed *in vitro *from pCDM8 plasmids using mMessenger mMachineTM kit (Ambion Inc., Austin, TX) according to manufacturer's instructions. The θ and ε clones were from Ewen Kirkness (The Institute for Genomics Research, Rockville, USA) and α3, β1 and γ2S clones from Paul Whiting (MSD Research Laboratories, Neuroscience Research Center, Essex, UK). Oocytes were removed during 0.2 % tricainemethanosulphonate (Sigma, St. Louis, MO) anesthesia from adult female *Xenopus laevis *frogs (Horst Kähler, Hamburg, Germany). Isolated oocytes were stored in Normal Frog Ringer (NFR containing in mM): 115 NaCl, 2.5 KCI, 18 CaCI_2 _and 10 HEPES; pH 7.5. Oocytes were then defolliculated manually with forceps and injected with 40 nl of a solution containing mixtures of subunit cRNAs (0.6 – 2.5 μg/μl) or pure water. Oocyte injections were carried out using a Drummond Nanoject injector (Drummond Scientific Co., Broomall, PA) via a glass micropipette having the tip diameter of about 17–20 μm. Subtype-specific cRNA cocktails were mixed beforehand, cRNA ratios of various subunits were 1:1 for α3β1 receptors, α3β1γ2S (1:1:10), α3β1θ (1:1:10), α3β1ε (1:1:1) and α3β1θε (1:1:10:1), one part being equivalent to the cRNA concentration of 2.5 μg/μl. A lower amount of ε cRNA than the γ2 and θ cRNA was injected, since higher amounts caused the oocytes to become leaky and unsuitable for electrophysiology. Oocytes were incubated at 18°C in incubation solution (MBS, Modified Barth Solution, supplemented with 2 mM sodium pyruvate, penicillin 100 U/ml and streptomycin 100 μg/ml). After injection (two h – one day) oocytes were digested for 30 min in Ca^2+^- free medium (82.5 mM NaCI, 2.5 mM KCI, 1 mM MgCI_2_, 1 mM Na_2_HPO_4 _and 5 mM HEPES; pH 7.5) containing 0.3 U/ml collagenase type IA (Sigma) in order to remove additional vitelline membrane surrounding the oocytes. After enzyme treatment, the oocytes were kept at 18°C in incubation solution until recordings.

### Electrophysiological recordings

Electrophysiological recordings were started 18 h after cRNA injection. Oocytes were perfused with NFR ± drugs at the flow rate of 1 ml/min in OPC-3 stand chamber (BioScience Tools, San Diego, US) at room temperature (22°C) using two-electrode voltage clamp, Ismatec pump (Ismatec, Glattbrugg-Zürich, Switzerland) and a 17-channel perfusion system with electronically controlled pinch valves as described [51]. Drug combinations were mixed prior to experiments. Oocytes were impaled with two microelectrodes that had resistance of 1.0–2.5 MΩ when filled with 3 M KCI plus 10 mM EGTA, and voltage clamped at -50 mV using Turbo TEC-05 two-electrode voltage-clamp amplifier (npi Electronics, Tamm, Germany). GABA, flurazepam hydrochloride and sodium pentobarbital (Sigma) were dissolved in NFR as 10 mM stock solutions. Etomidate (Sigma) and propofol (Tocris Cookson, Avonmouth, UK) were dissolved into ethanol (final concentration < 0.1 %) or dimethylsulphoxide (DMSO, maximal concentration = 0.3 % by vol.), respectively. 5α-Pregnan-3-α-ol-20-one (Sigma) was prepared as 10 mM stock in DMSO and maximal final vehicle concentration of DMSO was 0.3 % (v/v) which had no effect alone or on GABA-elicited currents (not shown). The pH of stock solutions was adjusted to 7.5. Gaboxadol (4,5,6,7-tetrahydroisoxazolo [5,4-c]pyridin-3-ol; THIP) was kindly provided by Bjarke Ebert (H. Lundbeck A/S, Copenhagen, Denmark). Experiments were controlled by EggWorks experimental control and data acquisition software program (version 3.0.2, npi). Drugs were applied for 10 s, and 180–300 s washout periods were used depending on drug concentrations and receptor sensitivity profiles. At least two different batches of oocytes were used to collect data for each analysis.

### Data analysis

Data analysis was performed using EggWorks Reader (version 3.0.2, npi) and Prism (version 3.0, GraphPad Software, San Diego, CA) programs. The amplitudes of peak currents were measured from recorded traces, normalized and presented as a percentage of the control current. The GABA dose-response curves (using the top and bottom fixed to 100 and 0, respectively) and some of the drug-effect curves (no fixed parameters) were analyzed using a sigmoidal nonlinear regression fit to estimate the EC_50 _values and maximal efficacies. To compare GABA sensitivity or drug effects on different GABA_A _receptor subtypes, one-way analysis of variance (ANOVA) and Dunnett's post test were used. The data are given as means ± standard errors.

## Authors' contributions

MR carried out the experiments and drafted the manuscript. STS helped with the planning of the experiments and the analyses of the experimental results. TM set up the electrophysiological method. MUO set up the methods for cRNA production. ERK planned the experiments and helped to interpret the results. All authors read and approved the final manuscript.
